# Prevention practices influencing frequency of occurrence of vaso-occlusive crisis among sickle cell patients in Abeokuta South Local Government Area of Ogun State, Nigeria

**DOI:** 10.1186/s12878-017-0077-9

**Published:** 2017-04-20

**Authors:** Olorunfemi Emmanuel Amoran, Ahmed Babatunde Jimoh, Omotola Ojo, Temitope Kuponiyi

**Affiliations:** 1 Department of Community Medicine and Primary Care, Olabisi Onabanjo University Teaching Hospital, Sagamu, Nigeria; 2 State Hospital Ala, Sagamu, Nigeria; 3 Department of Heamatology, College of Health Sciences, Olabisi Onabanjo University Teaching Hospital, Sagamu, Nigeria

## Abstract

**Background:**

Africa is the most affected continent with 200,000 new born affected by sickle cell anemia annually with of 5% of under five deaths. Nigeria has the largest sickle cell gene pool in the world with about 2% of all babies born to Nigerian parents. This study therefore sets out to assess the prevention practices influencing the frequency of occurrence of vaso-occlusive crisis among patients in Ogun State.

**Methods:**

This study is a descriptive cross-sectional study conducted in Abeokuta South Local Government Area Ogun State. A consecutive non randomized sampling of all the sickle cell patients that attend the selected facilities was recruited into the study. Data were collected with the use of questionnaires which were interviewer administered. A total of 415 patients were recruited into the study. Statistical analyses were conducted using SPSS for Windows version 20.0.

**Result:**

Two- third [64.8%] of study participants have crisis twice or more in a month. The frequency of crisis was statistically significantly associated with the age of the child [*p* = 0.006], use of anti-malaria prophylaxis [*p* = 0.006], analgesics [*p* = 0.0001], taking of plenty fluid [*p* = 0.001] and soothing herbs [*p* = 0.0001]. Lifestyle factors such as giving balance diet [*p* = 0.217], restriction from strenuous activities [*p* = 0.08], and attending Clinic appointments regularly [*p* = 0.126] were not statistically associated with reduction in the frequency of crisis. Logistic regression analysis shows that predictors of frequent crisis were individuals who were using prophylaxis antimalarial drugs [OR = 0.12, CI = 0.05–0.33] and analgesics [OR = 0.15, C.I = 0.06–0.34].

**Conclusion:**

The study reveals that majority of the participants have high frequency of crisis in a month. Drug prophylaxis rather than lifestyle factors may be more important in the prevention of vaso-occlusive crisis among sickle cell patients.

**Electronic supplementary material:**

The online version of this article (doi:10.1186/s12878-017-0077-9) contains supplementary material, which is available to authorized users.

## Background

Sickle cell anaemia contributes the equivalent of 5% of under five deaths on the African continent, more than 9% of such deaths in west Africa, and up to 16% of under-five deaths in individual west African countries [1]. Africa is the most affected continent with 200,000 new born affected by sickle cell anemia per year. This constitutes approximately 66.6% of the children born with haemoglobinopathies worldwide [2]. Nigeria has the largest sickle cell gene pool in the world. The sickle cell trait prevalence in Nigeria ranges from 25 to 35%. About 2% of all babies born to Nigerian parents have sickle cell anaemia. Two per hundred births translates to over 150,000 births annually of children with sickle cell anaemia [1]. The prevalence of SCD in Uganda is believed to be the highest in the whole world and it accounts for approximately 16.2% of all pediatric deaths [2].

Appropriate prevention practices and proper management will lead to reduction in the frequency of vaso-occlusive crisis. SCD patients presents with symptoms such as vaso-occlusive pain crises, anemia, dactylitis or hand-foot syndrome, eye damage, splenic sequestration etc. [3–6]. These symptoms occurs in SCD patients as a result of blood cells sticking to the walls of the blood vessels in the brain limiting blood flow [7]. Timely intervention and appropriate prevention practices is essential in the prevention of complications of vaso-occlusive crisis [8–16].

Children with SCD need optimal family support, understanding and care, especially in terms of providing adequate nutrition and health care delivery so as to achieve an optimum and steady state of health. Such favorable family environment and appropriate prevention measures has been shown to be a good prognostic index [17, 18]. The psychosocial burden and stress parents of sickle cell patients undergo could influence their attitude towards the care of their children positively or negatively. This study therefore sets out to assess the prevention practices influencing the frequency of occurrence of vaso-occlusive crisis among patients in Ogun State… Their knowledge and practices towards reducing the frequency and seeking appropriate treatment of vaso-occlusive crisis in their children is inevitable and also help in improving the quality of life of these children.

## Methods

### Study location and population

Abeokuta South is a Local Government Area in Ogun State, Nigeria. It was established in 1991 and mainly inhabited by the Egbas, who are of Egba Eku, Egba Aarin and Egba Igbeyin. The headquarter of the LGA is at Abeokuta7°09′00″N 3°21′00″E. It has an area of 71 km^2^ and a population of 250,278 at the 2006 census. The Local Government shares border with Odede LGA on its North frontier, Obafemi/Owode on the Eastern while Abeokuta North LGA on the Southern part respectively. The Local Government is divided into 15wards for the purpose of electing councilors into the Local Government Council. Each electoral ward has primary health centre, private clinics, laboratories, pharmacy shops, and traditional birth attendants.

### Study design

This study is a descriptive cross-sectional study to describe systematically the prevention practices influencing the frequency of occurrence of vaso-occlusive crisis among patients Abeokuta south Local Government Area of Ogun State, Nigeria.

#### Setting

This study was conducted in State Hospital Sokenu, Abeokuta and Egba Medical Centre, Isabo, Abeokuta both in Abeokuta South Local Government Area. The two hospitals are the two major sickle cell treatment centre in Abeokuta South LGA, Ogun State.

### Sampling technique

A consecutive non randomized sampling of all the sickle cell patients that attend the selected facilities was used to recruit participants into the study.

#### Sample size determination

The minimum number of subjects required for the study is calculated using the formula :

Minimum sample size, *n* = Z2 p q/d2

Where Z is the standard deviation set at 1.96 at 95% confidence interval, *p* = prevalence set at 50%, q = 1-p, and d = degree of accuracy set at 0.05

$$ \begin{array}{l} n = Z2\ \mathrm{pq}/\mathrm{d}2\\ {}\frac{=1.96\ 2\ \mathrm{x}0.5\mathrm{x}0.5}{0.05^2 = 384.}\end{array} $$

n was calculated to be 384.

A total of 415 participants were recruited.

### Data collection method

Data were collected with the use of interviewer administered pre-validated questionnaire. The interviewers were volunteer Doctors, Nurses and Laboratory Scientists. The interviewers were previously briefed on the nature and significance of the study and they were trained on how to administer the questionnaire under supervision. On the clinic days of each selected health facility, the researchers meet the participants, re- explained the purpose of the study and assured them of confidentiality of privileged information and a feedback after the study as told to them in the last meeting. The study was conducted between January and April 2015.

### Ethical approval

Ethical approval to conduct the study was obtained from the ethical committee of the Olabisi Onabanjo University Teaching Hospital, Sagamu. Permission was also obtained from the selected health facilities for the study, discussed with the Officers in-charge on the aims and objectives of the study, the procedure and feedback was assured which could contribute to the management of their patients. At the end of each meeting with the hospital management, verbal consent was obtained.

Written consent was obtained from the study participants before the commencement of the study. The participants were met on the day of clinics by the researchers and the purpose, general content and nature of the study were explained and they were given assurance of confidentiality of information offered and feedback at the end of the study.

For participants under the age of 16, written parental consent was obtained. All the patients have their parents or guardians present in the clinic with them to care for them.

### Data analysis

Statistical analyses were conducted using SPSS for Windows version 20.0 First, descriptive statistics were generated for each survey measure. Quantitative data collected was checked for errors, cleaned and entered. Data was summarized with proportions and means and presented using frequency tables. Frequency of crisis was categorized as either Once or less monthly (low frequency) and twice or more monthly as high frequency of crisis.

Variables such as plenty fluid was elicited by asking how many glass of cup of water do you take daily- showing them a 500mls cup. Three litres [6 glass of cup daily] was classified as adequate. Balance diet was taken as the description of diet containing carbohydrate, protein and fats for three previous meals. A strenuous activity was described as patient’s involvement in additional non routine, high oxygen demanding activities.

Bivariate analyses using the *X* ^2^ test were used to compare the socio-demographics of participants with the frequency of sickle cell crisis status of their child. The level of statistical significance was set at 5%. A logistic regression model was produced with low and high frequency of crisis as outcome variable. All explanatory variables that were associated with the outcome variable in bivariate analyses, variables with a *P*-value of ≤0.05 were included in the logistic models.

## Results

### Socio-dermographic characteristics of study participants

A total of 415 patients were recruited into the study. About half 42.7% of participants’ with SCD age is between 5 and 9 years, 29.4% were under-fives, 22.4% were adolescents aged between 10 and 19 years, 4.8% were young adults between 20 and 24 years and 0.7% of them were 25 years and above. Sex distribution of the children with SCD showed that 53.5% were male and 46.5% females. A third [35.2%] have crisis once or less in a month, 46% have crisis twice in a month, 18.8% have crisis thrice or more in a month (Table 1).

**Table 1:** Demographic Characteristics of study Participants

VARIABLES	FREQUENCY (*n* = 415)	PERCENTAGE (%)
Age of Participants with SCD		
1–4 years	122	29.4
5–9 years	177	42.7
10–14 years	66	15.9
15–19 years	27	6.5
20–24 years	20	4.8
25 years & above	3	0.7
Total	415	100.0
Sex of Participants with SCD		
Male	222	53.5
Female	193	46.5
Total	415	100.0
Number of Siblings lost due to SCD		
One	62	14.9
Two	10	2.4
Three	4	1.0
Four	1	0.2
None	338	81.4
Total	415	100.0

### Practice of vaso-occlusive crisis prevention methods

Table 2 shows the distribution on the method of treating SCD crisis, 79% give pain relief drugs at home, 60.7% give anti-malarial, 5.3% patronize local chemist, 2.9% take them to traditional healers/missionary homes while 29.6% more use of herbal remedies, 93.0% parent takes the child to the hospital and 82.4% of participant often meet doctor on duty, 15.9% reasonably often. Table 3 shows the distribution of participants on the method of preventing crisis, 77.3% give plenty of fluids, 73.5% take balance diet, 66.3% use insecticide treated net, 53.7% give prophylaxis drugs, 64.8% dress child in warm clothing, 45.5% restrict child from strenuous oxygen demanding activity, 2.9% sought spiritual means and 42.7% keep clinic appointments whether there’s crisis or not.

**Table 2:** Practice of vaso-occlusive crisis Prevention Methods

METHODS OF PREVENTING SC CRISIS	FREQUENCY (*n* = 415)	PERCENTAGE (%)
Giving Plenty Fluids		
Yes	321	77.3
No	94	22.7
Total	415	100.0
Giving Balance diet		
Yes	305	73.5
No	110	26.5
Total	415	100.0
Ensure Sleeping Under ITN		
Yes	275	66.3
No	140	33.7
Total	415	100.0
Giving Prophylactic Drugs		
Yes	223	53.7
No	192	46.3
Total	415	100.0
Dressed in Warm Clothing		
Yes	269	64.8
No	146	35.2
Total	415	100.0
Restriction from Strenuous Activity		
Yes	189	45.5
No	226	54.5
Total	415	100.0
Keep Clinic Appointments		
Yes	177	42.7
No	238	57.3
Total	415	100.0
Sought Spiritual Means		
Yes	12	2.9
No	403	97.1
Total	415	100.0

**Table 3:** Use of Prophylaxis in Prevention of vaso-occlusive crisis

METHOD OF TREATING SC CRISIS	FREQUENCY (*n* = 415)	PERCENTAGE (%)
Give Pain Relieving Drugs at Home		
Yes	328	79.0
No	87	21.0
Total	415	100.0
Give Anti-malaria Drug at Home		
Yes	252	60.7
No	163	39.3
Total	415	100.0
Take Child to Nearby Chemist		
Yes	22	5.3
No	393	94.7
Total	415	100.0
Take Child to Traditional healer/Missionary home		
Yes	12	2.9
No	403	97.1
Total	415	100.0
Give Local Herbal Remedies at Home		
Yes	123	29.6
No	292	70.4
Total	415	100.0
Take Child to The Hospital		
Yes	386	93.0
No	29	7.0
Total	415	100.0
How often they meet Doctor on duty		
Often	342	82.4
Reasonably often	66	15.9
Not often	7	1.7
Total	415	100.0

### Factors associated with frequency of crisis

The frequency of vaso-occlusive crisis was statistically significantly associated with the age of the participant with SCD [*X* ^2^ = 16.412, *p* = 0.006] and use of anti-malaria prophylactics [*X* ^2^ = 7.697, *p* = 0.006], analgesics [*X* ^2^ = 29.186, *p* = 0.0001], and soothing herbs [*X* ^2^ = 16.918, *p* = 0.0001]. There was however no statistically significant relationship between frequency of vaso-occlusive crisis and the Sex of the child with SCD [*X* ^2^ = 16.412, *p* = 0.06], and the number of siblings with SCD [*X* ^2^ = 7.086, *p* = 0.131].

Lifestyle factors such as giving balance diet [*X* ^2^ = 1.524, *p* = 0.217], Restriction from strenuous activities [*X* ^2^ = 3.072, *p* = 0.08], and attending clinic appointments regularly [*X* ^2^ = 2.345, *p* = 0.126] were not statistically associated with reduction in the frequency of crisis. While taking of plenty of fluid was statistically significantly associated with low frequency of crisis [*X* ^2^ = 13.44, *p* = 0.001]. This is as shown in Table 4.

**Table 4:** Factors associated with frequency of vaso-occlusive crisis

GIVING PLENTY FLUIDS	FREQUENCY OF CRISIS IN A MONTH		TOTAL
	LOW	HIGH	
YES	98(67.1%)	223(82.9%)	321(77.3%)
NO	48(32.9%)	46(17.1%)	94(22.7%)
TOTAL	146(100.0%)	269(100.0%)	415(100.0%)
FEEDING WITH BALANCE DIET			
YES	102(69.9%)	203(75.5%)	305(73.5%)
NO	44(30.1%)	66(24.5%)	110(26.5%)
TOTAL	146(100.0%)	269(100.0%)	415(100.0%)
\USE OF ITN			
YES	89(61.0%)	186(69.1%)	275(66.3%) 0.092
NO	57(39.0%)	83(30.9%)	140(33.7%)
TOTAL	146(100.0%)	269(100.0%)	415(100.0%)
USE OF PROPHYLAXIS DRUGS			
YES	65(44.5%)	158(58.7%)	223(53.7%) 0.006
NO	81(55.5%)	111(41.3%)	192(46.3%)
TOTAL	146(100.0%)	269(100.0%)	415(100.0%)
DRESSED IN WARM CLOTHING			
YES	98(67.1%)	171(63.6%)	269(64.8%) 0.479
NO	48(32.9%)	98(36.4%)	146(35.2%)
TOTAL	146(100.0%)	269(100.0%)	415(100.0%)
RESTRICTION FROM STRENOUS ACTIVITY			
YES	58(39.7%)	131(48.7%)	189(45.5%) 0.08
NO	88(60.3%)	138(51.3%)	226(54.5%)
TOTAL	146(100.0%)	269(100.0%)	415(100.0%)
GIVING ANTI-MALARIA DRUGS AT HOME			
YES	69(47.3%)	183(68.0%)	252(60.7%) .0001
NO	77(52.7%)	63(32.0%)	163(39.3%)
TOTAL	146(100.0%)	269(100.0%)	415(100.0%)
TAKEN TO THE HOSPITAL			
YES	132(90.4%)	254(94.4%)	386(93.0%) 0.126
NO	14(9.6%)	15(5.6%)	29(7.0%)
TOTAL	146(100.0%)	269(100.0%)	415(100.0%)
USE OF LOCAL HERBAL REMEDIES			
YES	25(17.1%)	98(36.4%)	123(29.6%) 0.0001
NO	121(82.9%)	171(63.6%)	292(70.4%)
TOTAL	146(100.0)	269(100.0%)	415(100.0%)
USE OF ANALGESICS AT HOME			
YES	94(64.4%)	234(87.0%)	328(79.0%) 0.001
NO	52(35.6%)	35(13.0%)	87(21.0%)
TOTAL	146(100.0%)	269(100.0%)	415(100.0%)

In the multiple logistic regression models, two variables were found to be independently associated with frequency of crisis. Predictors of low frequent crisis were individuals who were using prophylaxis antimalarial drugs [OR = 0.12, CI = 0.05–0.33] and analgesics [OR = 0.15, C.I = 0.06–0.34]. Herbs [OR = 3.74, C.I = 0.71–19.73], age of child [OR = 0.063, C.I = 0.001–4.11] and taking plenty of fluid [OR = 1.02, C.I = 0.27–3.78] were not predictors. This is shown in Table 5.

**Table 5:** Multivariate logistic Regression

	Odds Ratio [C.I]
Age of Participants	
Nil	1.00
Primary	0.063 [0.001–4.11]
Secondary	0.11 [0.002–6.63]
Tertiary	0.08 [0.001–5,47]
Antimalarial prophylaxis	
Yes	0.122 [0.05–0.33]
No	1.00
Analgesics	
Yes	0.145 [0.06–0.34]
No	1.00
Herbal use	
Yes	3.74 [0.71–19.73]
No	1.00
Taking Plenty of fluid	
Yes	1.02 [0.27–3.78]
No	1.00

## Discussion

The study reveals that majority of the participants have high frequency of crisis in a month despite adequate knowledge in the prevention of crisis and predisposing factors. The high frequency of sickle cell crisis amongst the participants is similar to what has been reported in previous studies [17–19] which reveals that there is overall increase in frequency of vaso occlusive crisis in paediatric SCD that its associated with poorer paediatric quality of life and increases in the psychosocial maladjustment of their caregivers. Obviously, knowledge does not translate to practice, however the prevention of vaso occlusive crisis requires huge financial capability which most of the parents cannot afford.

The age of the participants with SCD shows significant influence in the frequency of crisis, those participants in older age group shows significant proportion having low frequency of crisis monthly as they are probably more mature to understand the cause and consequence of the disease, follow rules and regulations,use their drugs when due and show the appropriate attitude towards preventing the frequency of crisis [15, 16]. They are able to detect and complain earlier about the onset of crisis and even seek appropriate treatment before it becomes severe.

However, more than half of the participants have adequate knowledge of how to prevent vaso-occlusive crisis. In this study majority had the practice of giving balance diet, giving plenty of fluid to prevent dehydration, use of analgesic and taking the patient to the hospital when severe. Over 90% of the participant knows that crisis could not be managed by chemist (road side drug sellers). Several studies have highlighted the fact that in Africa parents and patients have inadequate information on how to prevent vaso-occlusive crisis [1, 20]. This is an indication that parents/caregivers need more information on the disease and there is need for health education to be intensified. A vigorous enlightenment campaign on sickle cell disease should be put in place through appropriate media like print and electronics. National Sickle Cell Centre should be developed to facilitate the development of National control programme, which will be integrated with the National Health Service.

The patronage of traditional healers and missionary homes and the use of local herbs have influence in the frequency of crisis as majority of the participants who use local herbs or seek treatment at the traditional healers have high frequency of sickle cell crisis. This can further worsen the QOL of the SCD child as the herbs given could accelerates the damage to major organs there by leading to increase in morbidity and mortality. The cultural values and believe of the people play a major role in their belief about the cause and form of treatment of diseases generally and SCD is not an exception. Several studies [21–23] have concluded that cultural beliefs have an influence on health and that illness can be caused by natural, preternatural and mystical factors. The preternatural explanation is related to belief in witchcraft where the onset of illness is attributed to the evil machination of an enemy and in most cultures, there is belief that sorcerer, wizard and malevolent human being can cause illness, including sickle cell disease. If parents believe their child is bewitched they are not likely to come to the hospital or follow medical advice if they do.

This study shows that drug prophylaxis rather than lifestyle factors modification may be more important in the reduction of frequency of vaso-occlusive crisis. Several studies have highlighted the importance of both drug prophylaxis and lifestyle modification in the prevention of vaso-occlusive crisis among sickle cell patients [8–14] Further experimental studies will be needed to ascertain which of this is more essential in the prevention of vaso-occlusive crisis but ethical issues in the conduct of such experimental studies will be a serious limitation. However, medical treatment of sickle cell disease should be highly subsidized to make it affordable and accessible for all. Counselling sessions should be encouraged in all treatment centres in order to reduce the burden of the disease.

## Conclusion

The high incidence of SCD in Nigeria makes it a public health burden. Overall knowledge about sickle cell disease is poor which makes it difficult to combat the outrageous disease. Health authorities and institutions should adopt measures that have been shown to be effective in the management of sickle cell disorder. More centres should be established for the management of SCD patients and more health personnels should be recruited to these centres for more effective control of the disease.

## Additional file

Additional file 1: Parental influence on survival among SCP new.sav. Parental influence on survival among Sickle cell patients. (SAV 112 kb)
